# Glucose Variability in People with Type 1 Diabetes: Associations with Body Weight, Body Composition, and Insulin Sensitivity

**DOI:** 10.3390/biomedicines12092006

**Published:** 2024-09-03

**Authors:** Julia F. Semenova, Anton Yu. Yushin, Anton I. Korbut, Vadim V. Klimontov

**Affiliations:** Laboratory of Endocrinology, Research Institute of Clinical and Experimental Lymphology—Branch of the Institute of Cytology and Genetics, Siberian Branch of Russian Academy of Sciences (RICEL—Branch of IC&G SB RAS), 630060 Novosibirsk, Russia; ekmxtyjr@yandex.ru (J.F.S.); antyush@yandex.ru (A.Y.Y.); korbutai@icgbio.ru (A.I.K.)

**Keywords:** type 1 diabetes, obesity, overweight, adiposity, insulin resistance, body composition, continuous glucose monitoring, time in range, glucose variability

## Abstract

The prevalence of overweight and obesity increases in people with type 1 diabetes (T1D). However, the impact of fat accumulation on glucose dynamics in T1D is poorly understood. We assessed continuous glucose monitoring (CGM) parameters in patients with T1D depending on their body weight, body composition, and insulin sensitivity. In 547 patients, including 238 overweight/obese individuals, CGM-derived time in range (TIR) and glucose variability (GV) were estimated. Body composition was assessed by DXA. Estimated glucose disposal rate (eGDR) was used as an indicator of insulin sensitivity. Overweight/obese patients, when compared to normal-weight ones, have a lower time below range (TBR) (<3 mmol/L), GV, and experienced fewer episodes of low glucose. In men, lower TIR, higher time above range (TAR), and GV reduction were associated with central adiposity assessed by total, trunk, and android fat mass. In women, gynoid fat mass only was associated with a lower TIR and higher TAR. The eGDR was a positive predictor of TIR and a negative predictor of TAR, TBR, and GV in men and women. In conclusion, adiposity in people with T1D is associated with a lower risk of CGM-confirmed hypoglycemia, higher TAR, and reduced GV. These features of daily glucose dynamics may be mediated by insulin resistance.

## 1. Introduction

Type 1 diabetes (T1D) is a chronic disease with a high risk of complications and a negative impact on quality of life. In 2022, the total number of people living with T1D was estimated to be about 8.75 million worldwide [1]. Life expectancy of a 10-year-old individual diagnosed with T1D varies from a mean of 13 years in low-income countries to 65 years in high-income countries [2].

Current research demonstrates some trends in the natural history and phenotype of T1D. Nowadays, new cases of the disease are more often registered in adults [1,2]. Another current feature of T1D is the presence of overweight or obesity in a significant proportion of patients [3,4]. In youths with T1D, the prevalence of overweight and obesity varies from 15 to 36% [4]. In this cohort, a higher body mass index (BMI) and waist circumference (WC) correlate with lower insulin sensitivity [5]. Metabolic syndrome is increasingly detected in patients with T1D [6,7]. A recent meta-analysis of 27 studies revealed that 23.7% of T1D individuals are affected by metabolic syndrome [8].

It can be assumed that reduced insulin sensitivity in overweight and obese patients with T1D may affect their insulin requirement, the quality of glycemic control, and the risk of complications. Indeed, it was shown that an increased BMI in people with T1D is associated with a higher insulin dose and a worse cardiometabolic risk factor profile [9]. In the Diabetes Control and Complication Trial (DCCT), higher insulin resistance at baseline was associated with an increased subsequent risk of micro- and macrovascular diseases [10]. In the Diabetes Control and Complications Trial/Epidemiology of Diabetes Interventions and Complications (DCCT/EDIC) study, high body weight variability in people with T1D was related to an increased risk of major adverse events and all-cause mortality [11].

Accumulating evidence suggests that body weight and fat mass could be associated with both mean glucose and glucose variability (GV) levels across different stages of the metabolic continuum. In subjects with normal glucose tolerance, those who are overweight demonstrated slightly higher mean glucose estimated by continuous glucose monitoring (CGM) than individuals with normal weight; both fat mass and fat distribution were associated with mean glucose and amplitude-dependent GV parameters [12]. Results of a recent meta-analysis including 71 studies indicate that, in people with prediabetes, GV is increased, but is less clearly associated with adiposity and insulin sensitivity [13]. In patients with T1D, the presence of metabolic syndrome was reported to be associated with higher GV [14]. It was shown that GV is related to the adverse vascular profile in T1D patients, but only in those with concomitant insulin resistance [15].

Currently, there is very little data on the glucose dynamics in overweight and obese people with T1D, as well as on the relationships between GV and body composition in this cohort. Therefore, in this study, we examined CGM-derived time in the glucose ranges and a number of GV parameters in patients with T1D depending on body weight, body composition, and insulin sensitivity.

## 2. Materials and Methods

### 2.1. Design

Adult Caucasian men and women with insulin-treated T1D were included in this cross-sectional single-center comparative study. The list of exclusion criteria included: age under 18 years, type 2 diabetes (T2D) or other specific types of the disease, current hyperglycemic crises (diabetic ketoacidosis or hyperglycemic hyperosmolar states), pregnancy, end-stage renal disease, malignancies, and accompanied diseases, which may affect glucose metabolism. Since most patients also participated in the study of inflammatory markers [16], chronic inflammatory or autoimmune diseases, acute infections within three months prior to the study, treatment with glucocorticoids, non-steroidal anti-inflammatory drugs, or immunosuppressive agents also applied as exclusion criteria.

The search for potentially eligible patients was performed using an institutional database. After matching the inclusion and exclusion criteria, 570 individuals with T1D were included in the study. Patients with a BMI < 25 kg/m^2^ and those with a BMI ≥ 25 kg/m^2^ were considered as two separate groups.

Real-time CGM data for the last week were used for the calculation of mean glucose, time in the glucose ranges, and GV parameters. Insulin sensitivity was estimated indirectly by the estimated glucose disposal rate (eGDR). About a quarter of the study participants (*n* = 149) underwent dual-energy X-ray absorptiometry (DXA) to assess body composition parameters.

**Figure 1:**
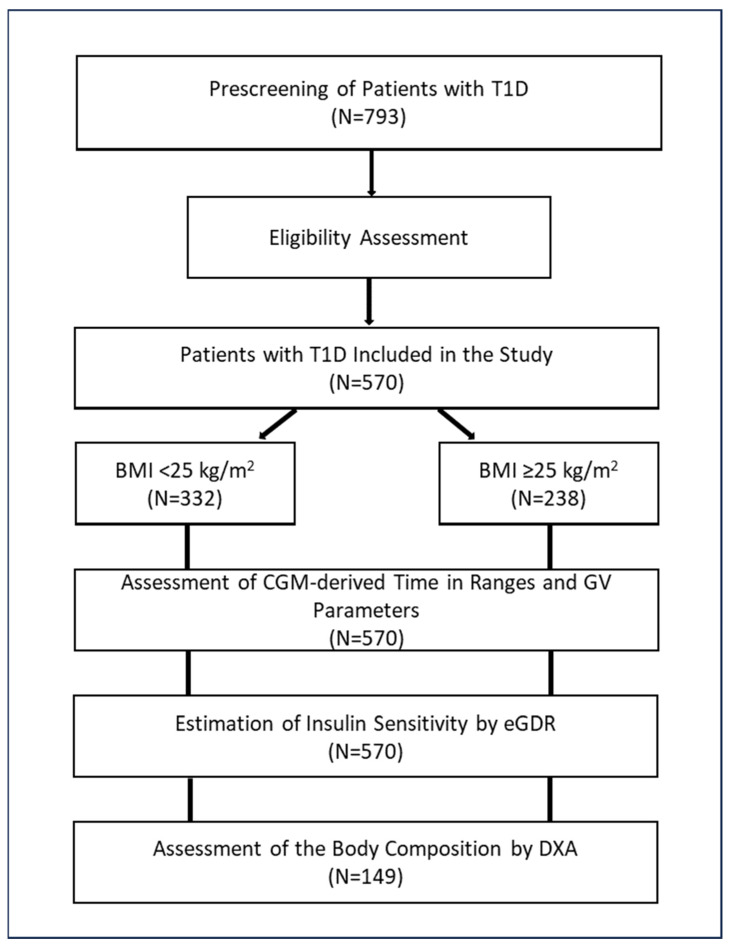
Study design. Abbreviations: BMI, body mass index; CGM, continuous glucose monitoring; DXA, dual-energy X-ray absorptiometry; eGDR, estimated glucose disposal rate; GV, glucose variability; T1D, type 1 diabetes.

The study design is shown in Figure 1.

### 2.2. Methods

All patients underwent a detailed clinical examination with an assessment of metabolic status and screening/monitoring of complications.

Real-time CGM was performed with MMT-722 or MMT-754 monitoring systems, Enlite sensors, and CareLink^®^ Pro software (2.5.524.0, v. 2.5A, Medtronic, Minneapolis, MN, USA). Patients were instructed to calibrate the systems at least 4 times a day. Median CGM duration was 6.7 days.

Time In the target Range (TIR: 3.9–10.0 mmol/L [70–180 mg/dL]); Time Above Range, Level 1 (TAR L-1: 10.0–13.9 mmol/L [180–250 mg/dL]); Time Above Range, Level 2 (TAR L-2: >13.9 mmol/L [>250 mg/dL]); Time Below Range, Level 1 (TBR L-1: 3.8–3.0 mmol/L, [< 70–54 mg/dL]); and Time Below Range, Level 2 (TBR L-2: <3.0 mmol/L [<54 mg/dL]) were estimated according to the international guidelines [17,18].

The following GV indices were calculated: Coefficient of Variation (CV), Mean Amplitude of Glycemic Excursions (MAGE), Mean Absolute Glucose rate of change (MAG), and Lability Index (LI). Characteristics of these indices can be found in recent reviews [19,20]. In brief, CV is a measure of the dispersion of glucose values, MAGE is the mean differences from peaks to nadirs, and MAG and LI estimate the rate of change in glucose concentration. The GV parameters were estimated with the use of an EasyGV v. 9.0.R2 calculator [21].

Mean numbers of glucose episodes of <3.9 mmol/L (<70 mg/dL) and <3.0 mmol/L (<54 mg/dL) per 24 CGM hours were analyzed also. 

eGDR was calculated with the use of the following formula: eGDR = 21.158 − (0.09 × WC) − (3.407 × hypertension) − (0.551 × HbA1c), where WC is the waist circumference (cm), hypertension (yes = 1/no = 0), and HbA1c = HbA1c (%) [22].

Body composition parameters were assessed by DXA with the use a Lunar Prodigy Advance densitometer and Body Composition software (GE healthcare, Madison, WI, USA). Specifically, we estimated total, trunk, android, gynoid fat masses, and lean mass, as described previously [23].

### 2.3. Statistical Analysis 

Statistical analysis was performed using the STATISTICA 12 software package (Dell, Round Rock, TX, USA). We used the Kolmogorov–Smirnov criterion to test normality. The differences between the groups in quantitative parameters were assessed using the Mann–Whitney test. Spearman’s rank correlation analysis and multiple stepwise regression analysis with a forward selection of variables were applied to test the associations between parameters. Medians, 25th and 75th percentiles, or minimum and maximum values are reported.

## 3. Results

### 3.1. Clinical Characteristics of Patients

The study included 570 patients, 211 men and 359 women, ranging from 18 to 73 years of age (median: 36 years). Diabetes duration varied from one to 55 years (median: 16 years). All patients received basal–bolus therapy with insulin analogs. Four-hundred subjects were managed with multiple daily injections (MDIs) and 170 individuals received a continuous subcutaneous insulin infusion (CSII) with the use of insulin pumps (mostly, MMT-722 or MMT-754, Medtronic, Minneapolis, MN, USA). Diabetic complications and/or associated diseases were verified in most patients: chronic kidney disease (*n* = 349), neuropathy (*n* = 349), diabetic retinopathy (*n* = 317), arterial hypertension (*n* = 216), impaired awareness of hypoglycemia (*n* = 200), non-alcoholic fatty liver disease (*n* = 187), peripheral artery disease (*n* = 84), diabetic foot (*n* = 44), and coronary artery disease (*n* = 29).

Three-hundred-and-thirty-two individuals had a BMI < 25 kg/m^2^ and two-hundred-and-thirty-eight patients had a BMI ≥ 25 kg/m^2^. In the second group, 156 subjects were overweight (BMI: 25–29.9 kg/m^2^) and 82 subjects were obese (BMI ≥ 30 kg/m^2^). 

The clinical characteristics of the study groups are presented in Table 1. Patients with a BMI ≥ 25 kg/m^2^, when compared to those with a BMI < 25 kg/m^2^, were older, had a slightly longer diabetes duration, and were treated with higher insulin doses. Patients with a BMI ≥ 25 kg/m^2^ demonstrated higher levels of total and LDL-cholesterol, triglycerides, uric acid, high-sensitivity C-reactive protein (hsCRP), and a lower estimated glomerular filtration rate (eGFR). No significant differences in glycated hemoglobin A1c (HbA1c) and albuminuria levels were found between the groups. The values of eGDR were markedly lower in overweight/obese patients. 

### 3.2. CGM Parameters in T1D Patients Depending on BMI and WC

Mean CGM glucose and TIR did not differ significantly between patients with a BMI ≥ 25 kg/m^2^ and those with a BMI < 25 kg/m^2^ (Table 2). Overweight/obese patients tended to have a higher TAR L-1 and lower TBR L-1; meanwhile, TBR-L2 was significantly lower in this group. There were no significant differences between the groups in CV and MAGE values. At the same time, lower MAG and LI values were revealed in overweight/obese individuals.

Patients with a BMI ≥ 25 kg/m^2^ experienced fewer episodes of low glucose levels per day than those with a BMI < 25 kg/m^2^: 0.33 (0.0; 0.86) and 0.21 (0.0; 0.56) episodes of glucose at <3.9 mmol/L, *p* = 0.007, and 0.0 (0.0; 0.13) and 0.0 (0.0; 0.0) episodes of glucose at <3.0 mmol/L, respectively, *p* = 0.018. 

In the Spearman’s rank correlation analysis, TBR L-2, MAG, and LI demonstrated weak negative correlations with BMI (Table 3). When men and women were considered separately, BMI correlated negatively with MAGE, MAG, and LI in men. In women, BMI demonstrated weak negative correlations with TBR L-2 and MAG, and a positive correlation with TAR L-1. 

Similarly to BMI, WC showed weak negative correlations with TBR L-2, MAG, and LI (Table 4). In men, WC correlated negatively with TAR L-1, CV, MAGE, MAG, and LI, and was associated positively with TIR. In women, WC showed weak negative correlations with TBR L-1, TBR L-2, and MAG, and demonstrated positive weak correlations with TAR L-1 and TAR L-2. 

### 3.3. CGM Parameters in T1D Patients Depending on Body Composition 

The assessment of body composition was performed on 149 subjects, 70 men and 79 women. Subjects with a BMI ≥ 25 kg/m^2^ demonstrated higher total, trunk, android, and gynoid fat mass, as well as lean mass, when compared to those with a BMI < 25 kg/m^2^ (Table 4).

**Table 4:** Parameters of body composition in patients with T1D depending on the BMI.

Parameter	Groups of Patients		*p*
	BMI < 25 kg/m^2^ N = 85	BMI ≥ 25 kg/m^2^ N = 64	
Total fat mass, kg	18.7 (14.3; 23.1)	30.6 (25.1; 34.4)	<0.0001
Total fat mass, %	30.6 (24.6; 36.9)	36.7 (31.6; 40.6)	<0.0001
Trunk fat mass, kg	8.2 (6.2; 11.5)	16.5 (13.4; 19.5)	<0.0001
Android fat mass, kg	1.2 (0.8; 1.7)	2.7 (2.2; 3.5)	<0.0001
Gynoid fat mass, kg	3.5 (2,7; 4.4)	4.6 (3.9; 5.7)	<0.0001
Lean mass, kg	43.1 (38.9; 51.7)	59.5 (46.7; 63.6)	<0.0001

Data are presented as medians [25% percentile; 75% percentile]. Abbreviations: BMI, body mass index; T1D, type 1 diabetes.

The values of TIR, TAR, and TBR did not correlate with the body composition parameters (Table 5). At the same time, MAG and LI demonstrated weak negative correlations with total, trunk, and android fat mass. MAGE correlated negatively with trunk fat mass only. No correlations were found between CGM-derived parameters and lean mass. In men, TIR correlated positively with total and trunk fat mass; TAR L-2 demonstrated negative correlations with total, trunk, android, and gynoid fat mass. In women, a positive correlation between CV and gynoid fat mass was found. 

### 3.4. Relationships between CGM Parameters and eGDR

The values of eGDR correlated negatively with BMI (r = −0.62, *p* < 0.0001), WC (r = −0.73, *p* < 0.0001), total fat mass (r = −0.52, *p* < 0.0001), trunk fat mass and android fat mass (both r = −0.58, *p* < 0.0001), gynoid fat mass (r = −0.27, *p* = 0.0009), and lean mass (r = −0.41, *p* < 0.0001). 

The correlations between eGDR and CGM parameters are presented in Table 6. In men, eGDR demonstrated positive correlations with TBR L-1, TBR L-2, MAG, and LI; weak negative correlations with TAR L-1 and TAR L-2 were found. In women, eGDR was related to TIR and MAG positively, and with TAR L-1, TAR L-2, TBR L-1, and TBR L-2 negatively. In addition, we found positive correlations between eGDR and a daily number of episodes of glucose <3.9 and <3 mmol/L (r = 0.22 and r = 0.28, respectively, both *p* < 0.001). 

### 3.5. Multivariate Models

We assessed the associations of CGM metrics with clinical, anthropometric, body composition parameters, and eGDR in two sets of multivariate models. 

In the first set, age, BMI, WC, diabetes duration, daily insulin dose, eGFR, and eGDR were included as independent variables. The results are presented in Table 7. 

In men, WC turned out to be a predictor of TIR, TAR L-1, TAR L-2, MAGE, and LI. The values of eGDR were associated with TIR, TAR L-1, TAR L-2, TBR L-2, and MAGE. In these models, daily insulin dose predicted MAG. No reliable models were built for TBR L-1 and CV. 

In women, BMI was associated with TIR, TAR L-2, CV, MAGE, and LI. An association between WC and MAG was found. eGDR predicted TIR, TAR L-1, TAR L-2, TBR L-1, MAGE, and LI. Age predicted TIR and TAR L-2, and eGFR was associated with TBR L-1, CV, MAGE, LI, and LBGI. No significant model for TBR L-2 was generated. 

In the second set of multivariate models, BMI and WC were replaced by body composition parameters, namely total, trunk, android, gynoid fat mass, and lean mass. The results are summarized in Table 8.

In men, total fat mass was a negative predictor of TIR and LI, and a positive predictor of TAR L-2. Android fat mass was associated negatively with CV and MAGE. Trunk fat mass was a negative predictor of MAGE. eGDR demonstrated a positive association with MAG. Daily insulin dose was associated with TIR negatively, demonstrating positive relationships with TAR L-1 and TAR L-2. No reliable models were built for TBR L-1 and TBR L-2.

In women, gynoid fat mass was the negative predictor of TIR and positive predictor of TAR L-1 and TAR L-2. eGDF was associated negatively with TAR L-2 and demonstrated a positive association with TBR L-2. Daily insulin dose was associated positively with TBR L-1 and CV. We failed to generate models for MAGE and MAG. eGFR turned out to be the only predictor of LI. 

## 4. Discussion

In this study, we assessed the associations of body weight, body composition, and insulin sensitivity to CGM-derived time in ranges and GV parameters in patients with T1D. To our knowledge, this is the first study demonstrating the relationships of adiposity and reduced insulin sensitivity with characteristics of glucose dynamics in these patients. For the in-depth analysis of CGM data, we calculated time in ranges (TIRs: TAR L-1, TAR L-2, TBR L-1, and TBR L-2), as well as GV indices, which characterize the dispersion of glucose values (CVs), the amplitude of glucose fluctuations (MAGEs), and the rate of change in glucose concentrations (MAGs, LI), as well as the number of low glucose episodes. 

### 4.1. Main Findings

The principal finding of the study is an association of adiposity with a reduction in TIR, amplitude-dependent GV, and a risk of hypoglycemia. Specifically, we found that overweight/obese people with T1D when compared to those with BMI < 25 kg/m^2^ have lower TBR L-2 (<3 mmol/L), MAG, and LI values, and experience fewer episodes of low glucose despite the higher daily insulin dose. Both BMI and WC showed weak negative correlations with TBR L-2, MAG, and LI. 

Previously, an inverse correlation between BMI and CV in T1D patients was reported [24]. In youths with T1D, an association between BMI and hyperglycemic excursions, but not with CV and MAGE, was observed [25]. In disagreement with these data, a positive association was reported between hypoglycemia, CV, and BMI percentile in children and youths with T1D [26]. The discrepancy of the data may be explained by the different populations surveyed, as well as by the fact that BMI may not reflect the characteristics of body composition in obese individuals with different physical activities and sensitivity to insulin [27].

In this study, we tested the hypothesis that differences in body composition and insulin sensitivity may explain the patterns of glucose dynamics in T1D patients with overweight/obesity and normal body weight. Taking into account sex differences in weight and fat distribution, we analyzed men and women separately. When studying the relationships between CGM parameters and body composition, we identified correlations between total, trunk, android and gynoid fat mass with high glucose (TAR L-2), and amplitude-dependent GV parameters (CV, MAGE, MAG, and LI) in men. In the multivariate regression analysis, total fat mass was associated with high glucose (TAR L-2), while android fat mass turned out to be a predictor of CV and MAGE. In women, gynoid fat mass, but not other body composition parameters, correlated with TAR L-1 and CV, and predicted TIR and TAR in the multivariate model. Recently, Lipsky LM et al. reported an association between adiposity and TAR in youths with T1D. However, no relationships were found between body composition and GV estimated by MAGE and standard deviation [25]. 

A number of studies have shown that android fat or its ratio to gynoid fat is associated with decreased insulin sensitivity and related metabolic abnormalities. In subjects with normal glucose tolerance, android fat mass was positively associated with insulin resistance (HOMA-IR index) and demonstrated negative correlations with TBR and Low Blood Glucose Index [12]. Similarly, in obese children and adolescents, the android/gynoid ratio was related to HOMA-IR [28]. In young overweight males, preferential fat accumulation in the android area was associated with insulin resistance, impairment in lipid profile, and endothelial dysfunction [29]. A population-based study performed in Hangzhou, China, showed that android/gynoid ratio is positively associated with non-alcoholic fatty liver disease [30]. The latter, in turn, is closely related to insulin resistance [31].

In our study, we used eGDR as a validated score based on WC, hypertension, and HbA1c, which was introduced to measure insulin sensitivity in T1D [32,33]. A reduction in eGDR was found in overweight/obese patients indicating a decrease in insulin sensitivity. The eGDR values correlated negatively with BMI, WC, total, trunk, and android fat mass, and, to a lesser extent, gynoid fat mass. In agreement, Q. Zeng et al. reported negative associations of eGDR with visceral fat index and trunk fat mass in Chinese subjects with T1D [34]. In the multivariate models, we recognized eGDR to be a positive predictor of TIR and a negative predictor of TAR, TBR, and MAGE in men and women, and LI in women. Therefore, we can assume that the observed associations of body weight and fat mass accumulation with hyperglycemia and reduced GV are mediated through a decrease in insulin sensitivity. 

Currently, the relationships between insulin sensitivity, CGM profiles, and, especially, GV are not well understood. Recently, I. Clinck et al. reported higher CGM-assessed TAR in patients with T1D with the lowest eGDR. In the regression analysis, a higher eGDR was associated with a higher TIR and TBR and a lower TAR. Our data confirm these results. Having studied a larger number of GV parameters, we also found a negative association between eGDR and GV. However, in the cited work, insulin sensitivity was assessed not only using the eGDR, but also by a hyperinsulinemic–euglycemic clamp. Interestingly, no significant relationships between CGM parameters and measured glucose disposal rate were found [35]. It was assumed that the enhancing effect of insulin on glucose fluctuations can be mitigated by insulin resistance [36]. In agreement, a reciprocal relationship between HOMA-IR and GV was revealed in subjects with prediabetes [37].

The results of our study show that total, trunk, and android fat mass values are related to CGM parameters in men, but not in women. This finding may be explained by sex differences in the association between abdominal fat accumulation and insulin sensitivity [38]. In Chinese adults, android fat percentage was more closely associated with metabolic syndrome components in men. In women, android fat percentage and gynoid fat percentage showed opposite effects on the levels of triglycerides, HDL-cholesterol, and WC [39]. In subjects with newly diagnosed T2D and individuals with prediabetes, android fat positively correlated with HOMA-IR in men, but not in women [40]. In transgender individuals receiving gender-affirming hormone therapy, android fat more strongly correlated with insulin resistance compared to gynoid fat [41].

Pathogenetic links between adiposity, insulin resistance, and T1D could be multidirectional. Patients with T1D have specific risk factors that contribute to weight gain. These include intensive insulin therapy, subcutaneous insulin administration leading to peripheral hyperinsulinemia, repetitive episodes of hypoglycemia, and the consequences of a fear of hypoglycemia in the form of frequent snacking and decreased physical activity. Diabetes-specific psychosocial burden (stress, depression, decreased quality of life, lack of support, etc.) can exacerbate obesity. Obesity triggers lipotoxicity, mitochondrial dysfunction, adipose tissue dysfunction, gut microbiome imbalance, low-grade inflammation, and, ultimately, reduces insulin sensitivity. In its turn, obesity-driven insulin resistance increases the requirement for exogenous insulin, forming a vicious circle. At the same time, the accelerator hypothesis postulates that obesity and insulin resistance can lead to glucotoxicity and accelerated apoptosis of pancreatic β-cells via immune and inflammatory mechanisms. Finally, some people with T1D may be genetically predisposed to obesity and insulin resistance, especially when they have a family history of T2D [4,42].

### 4.2. Clinical Implications

An increasing prevalence of overweight and obesity in people with T1D is a serious challenge. Although patients with insulin resistance may have a reduced risk of hypoglycemia, other factors associated with decreased sensitivity to insulin may have a detrimental effect on vascular health and disease outcomes. A 10.9-year follow-up of 26,125 T1D patients registered in the Swedish National Diabetes Registry showed an increase in the risk of major cardiovascular disease, heart failure, cardiovascular death, and mortality with increasing BMI. These associations were more apparent in men than in women [43]. In subjects with T1D, insulin resistance verified by the eGDR was associated with vascular complications independently from HbA1c [44,45]. The results of recent meta-analysis demonstrate that insulin resistance, as estimated by the eGDR, may be an additional risk factor for cardiovascular disease and all-cause mortality in T1D [46]. Data from our and other studies [9,47] indicate that overweight and obese T1D patients require higher insulin doses for glycemic control than their lean counterparts, which may pose an additional risk of complications and impede weight loss. Therefore, the prevention of overweight and obesity should become a part of the management strategy for T1D patients as well.

The features of metabolic changes in obese patients with T1D should be taken into account in clinical practice. Our results indicate that individuals with T1D who are overweight or obese are less likely to achieve glycemic control targets, especially in managing hyperglycemia, than patients with normal weight. Therefore, adiposity-driven insulin resistance should be considered as a barrier to the control of T1D. Currently, there are several options to manage or even eliminate this barrier. Lifestyle modification programs, including nutrition therapy, correction of dietary habits, and increased levels of physical activity, should be an essential part of comprehensive care for people with T1D and obesity [48]. Glucagon-like peptide-1 receptor agonists, dual glucagon-like peptide-1 and gastric inhibitory polypeptide agonists, sodium-glucose cotransporter-2 inhibitors, metformin, pramlintide, and some other drugs are discussed as promising therapeutic agents for the management of obesity and/or insulin resistance in patients with T1D [49,50]. Bariatric surgery may be a viable treatment for patients with T1D and severe obesity [51]. However, it should be noted that drug therapy and surgical treatment are not yet standard approaches to weight management in T1D.

### 4.3. Limitations of the Study and Future Remarks

Limitations of our study include cross-sectional design, patient recruitment in one clinical center, and short CGM duration. We did not differentiate between visceral and subcutaneous adipose tissue when assessing body composition. Insulin sensitivity was estimated indirectly by eGDR, but not by a clamp technique. We did not take into account the level of physical activity, dietary habits, stress, and other lifestyle and psychosocial factors of the study participants. It is likely that these factors could modify some of the associations between GV, body composition, and insulin sensitivity we observed. The lack of a control group does not allow us to compare associations between body composition and glucose dynamics in people with T1D with those in healthy individuals. The representativeness of the sample is limited to young and middle-aged adults with T1D without advanced complications and serious concomitant diseases. In addition, the study focuses on a Caucasian population, which may limit the generalizability of the findings to other ethnic groups. 

The strengths of this study are the large sample size, comprehensive analysis of CGM data with a broad range of time-dependent and amplitude-dependent GV parameters, and the use of DXA to examine body composition. In an effort to provide insights into the whole story, we assessed glucose dynamics, anthropometric parameters, body composition, and insulin sensitivity in a sample of patients with T1D.

Future studies are needed for the identification of mechanisms underlying the associations of fat mass and fat distribution with glucose homeostasis in T1D patients. Prospective studies could answer the question of how changes in body weight and body composition affect insulin sensitivity and glucose dynamics in people living with T1D. In particular, the effect of weight loss and adiposity reduction on TIR and GV parameters in obese subjects with T1D is a great challenge.

## 5. Conclusions

The results of this study demonstrate that overweight and obese people with T1D have a lower risk of CGM-confirmed hypoglycemia and reduced GV than those with a BMI <25 kg/m^2^. In men, central adiposity assessed by the total, trunk, and android fat mass is associated with higher glucose and lower GV. In women, gynoid fat mass, but no other body composition parameters, is related to hyperglycemia. The observed associations of body weight and body composition with the features of daily glucose dynamics may be mediated by insulin sensitivity.

## Figures and Tables

**Table 1:** Clinical characteristics of study participants.

Characteristics	Groups of Patients with T1D		*p*
	BMI < 25 kg/m^2^ N = 332	BMI ≥ 25 kg/m^2^ N = 238	
Age, years	32 (25; 43)	41 (33; 54)	<0.0001
Sex, m/f, *n* (%)	96/236 (28.9/71.1)	115/123 (48.3/51.7)	<0.0001
BMI, kg/m^2^	21.8 (20.0; 23.3)	28.5 (26.6; 31.0)	<0.0001
WC, cm	75 (70; 80)	96 (87; 103)	<0.001
Diabetes duration, years	15 (8.5; 22)	17 (10; 26)	0.017
Daily insulin dose, IU	42 (32; 52)	58 (45; 74)	<0.0001
Daily insulin dose, IU/kg	0.7 (0.5; 0.9)	0.7 (0.6; 0.9)	0.476
MDIs/CSII, *n* (%)	220/112 (66.3/33.7)	180/58 (75.6/24.4)	0.0001
HbA1c, %	8.1 (7.0; 9.3)	8.1 (7.3; 9.1)	0.827
HbA1c, mmol/L	64.6 (53.1; 78.6)	64.9 (55.9; 76.2)	0.827
Total cholesterol, mmol/L	4.8 (4.1; 5.8)	5.2 (4.3; 5.9)	0.076
LDL-cholesterol, mmol/L	2.9 (2.4; 3.5)	3.2 (2.5; 3.9)	0.001
HDL-cholesterol, mmol/L	1.5 (1.3; 1.9)	1.4 (1.2; 1.6)	<0.0001
Triglycerides, mmol/L	0.93 (0.68; 1.27)	1.16 (0.86; 1.63)	<0.0001
Uric acid, μmol/L	233 (193; 287)	264 (222; 322)	<0.0001
hsCRP, mg/L	1.07 (0.52; 2.05)	2.12 (1.14; 4.22)	<0.0001
eGFR, mL/min/1.73 m^2^	91 (76; 105)	84 (71; 96)	0.0002
UACR, mg/mmol	2.2 (2.0; 5.89)	2.1 (2.0; 4.74)	0.307
eGDR, mg/kg/min	9.6 (7.2; 10.5)	5.3 (3.9; 7.9)	<0.0001

Continuous data are presented as medians (25% percentile; 75% percentile) and frequencies are presented as numbers of patients and percentages. Abbreviations: BMI, body mass index; eGDR, estimated glucose disposal rate; eGFR, estimated glomerular filtration rate; HbA1c, glycated hemoglobin A1c; HDL, high-density lipoprotein; hsCRP, high-sensitivity C-reactive protein; LDL, low-density lipoprotein; MDIs/CSII multiple daily injections/continuous subcutaneous insulin infusion; T1D, type 1 diabetes; UACR, urinary albumin-to-creatinine ratio; WC, waist circumference.

**Table 2:** CGM parameters in patients with T1D depending on BMI.

Parameter	Groups of Patients		*p*
	BMI < 25 kg/m^2^ N = 332	BMI ≥ 25 kg/m^2^ N = 238	
Mean glucose, mmol/L	7.9 (6.9; 9.2)	8.3 (7.2; 9.3)	0.143
TIR, %	74.5 (59.0; 85.3)	69.4 (58.6; 83.3)	0.423
TAR L-1, %	18.9 (9.3; 28.3)	21.1 (12.6; 30.4)	0.061
TAR L-2, %	2.6 (0.3; 9.3)	3.3 (0.5; 8.6)	0.389
TBR L-1, %	0.6 (0.0; 2.5)	0.6 (0.0; 1.7)	0.055
TBR L-2, %	0.0 (0.0; 0.2)	0.0 (0.0; 0.0)	0.017
CV, %	30.5 (27.0; 33.9)	29.6 (26.9; 33.8)	0.474
MAGE, mmol/L	3.9 (3.2; 4.8)	3.9 (3.3; 4.6)	0.299
MAG, mmol/L/h	2.1 (1.7; 2.5)	1.9 (1.7; 2.3)	0.001
LI, (mmol/L)^2^/h	3.2 (2.3; 4.7)	2.9 (2.2; 3.9)	0.013

Data are presented as medians [25% percentile; 75% percentile]. Abbreviations: BMI, body mass index; CGM, continuous glucose monitoring; CV, Coefficient of Variation; LI, Lability Index; MAG, Mean Absolute Glucose rate of change; MAGE, Mean Amplitude of Glycemic Excursions; TAR L-1, Time Above Range, Level 1; TAR L-2, Time Above Range, Level 2; TBR L-1, Time Below Range, Level 1; TBR L-2, Time Below Range, Level 2; TIR, Time In the target Range; T1D, type 1 diabetes.

**Table 3:** Correlations of CGM parameters with BMI and WC in patients with T1D.

Parameter	BMI			WC		
	All	Men	Women	All	Men	Women
TIR	−0.01	0.1	−0.08	0.0	0.2 *	−0.12
TAR L-1	0.06	−0.06	0.13 *	0.02	−0.17 *	0.15 *
TAR L-2	0.02	−0.09	0.08	0.03	−0.15	0.14 *
TBR L-1	−0.05	−0.03	−0.08	−0.08	−0.07	−0.14 *
TBR L-2	−0.11 **	−0.08	−0.12 *	−0.16 **	−0.11	−0.21 **
CV	−0.04	−0.13	0.0	−0.04	−0.17 *	−0.02
MAGE	−0.07	−0.18 **	−0.02	−0.08	−0.31 **	0.04
MAG	−0.18 **	−0.23 **	−0.15 **	−0.22 **	−0.37 **	−0.16 *
LI	−0.15 **	−0.24 **	−0.1	−0.17 **	−0.39 **	−0.06

Spearman’s rank correlation coefficients (r) are presented. Significant coefficients are highlighted in bold: * *p* < 0.05; ** *p* < 0.01. Abbreviations: BMI, body mass index; CGM, continuous glucose monitoring; CV, Coefficient of Variation; LI, Lability Index; MAG, Mean Absolute Glucose rate of change; MAGE, Mean Amplitude of Glycemic Excursions; TAR L-1, Time Above Range, Level 1; TAR L-2, Time Above Range, Level 2; TBR L-1, Time Below Range, Level 1; TBR L-2, Time Below Range, Level 2; TIR, Time In the target Range; T1D, type 1 diabetes; WC, waist circumference.

**Table 5:** Correlations between CGM metrics and body composition parameters in patients with T1D.

Parameter	TIR	TAR L-1	TAR L-2	TBR L-1	TBR L-2	CV	MAGE	MAG	LI
**All patients**									
Total fat mass	0.05	−0.06	−0.07	−0.04	−0.07	−0.12	−0.11	−0.22 **	−0.19 *
Trunk fat mass	0.09	−0.11	−0.11	−0.04	−0.06	−0.14	−0.17 *	−0.26 **	−0.24 **
Android fat mass	0.08	−0.09	−0.11	−0.06	−0.08	−0.15	−0.14	−0.26 **	−0.22 **
Gynoid fat mass	0.04	−0.05	−0.06	−0.06	−0.02	−0.11	−0.04	−0.17 *	−0.12
Lean mass	−0.01	0.01	−0.01	0.07	0.01	0.01	0.01	−0.1	−0.05
**Men**									
Total fat mass	0.27 *	−0.24 *	−0.37 **	−0.02	0.01	−0.27 *	−0.43 **	−0.36 **	−0.49 **
Trunk fat mass	0.25 *	−0.21	−0.34 **	−0.04	−0.03	−0.27 *	−0.45 **	−0.37 **	−0.49 **
Android fat mass	0.22	−0.18	−0.32 **	−0.04	−0.04	−0.27 *	−0.40 **	−0.35 **	−0.46 **
Gynoid fat mass	0.23	−0.21	−0.34 **	0.02	0.04	−0.23	−0.34 **	−0.29 *	−0.38 **
Lean mass	0.12	0.01	−0.19	−0.04	−0.08	−0.23	−0.22	−0.13	−0.25 *
**Women**									
Total fat mass	−0.16	0.21	−0.03	−0.13	0.14	0.18	−0.12	0.07	0.07
Trunk fat mass	−0.03	0.09	−0.06	−0.11	0.03	0.08	−0.17	−0.01	−0.01
Android fat mass	−0.03	0.07	−0.11	−0.16	0.02	0.09	−0.18	−0.02	−0.02
Gynoid fat mass	−0.19	0.24 *	−0.11	−0.07	0.19	0.23 *	−0.12	0.08	0.08
Lean mass	0.01	−0.01	−0.03	−0.06	−0.06	0.08	−0.12	−0.0	−0.0

Spearman’s rank correlation coefficients (r) are presented. Significant coefficients are highlighted in bold: * *p* < 0.05; ** *p* < 0.01. Abbreviations: CGM, continuous glucose monitoring; CV, Coefficient of Variation; LI, Lability Index; MAG, Mean Absolute Glucose rate of change; MAGE, Mean Amplitude of Glycemic Excursions; TAR L-1, Time Above Range, Level 1; TAR L-2, Time Above Range, Level 2; TBR L-1, Time Below Range, Level 1; TBR L-2, Time Below Range, Level 2; TIR, Time In the target Range; T1D, type 1 diabetes.

**Table 6:** Correlations of CGM parameters with eGDR in patients with T1D.

Parameter	All Patients	Men	Women
TIR	0.19 **	0.10	0.25 **
TAR L-1	−0.23 **	−0.14 *	−0.30 **
TAR L-2	−0.22 **	−0.14 *	−0.27 **
TBR L-1	0.25 **	0.25 **	−0.27 **
TBR L-2	0.24 **	0.22 **	−0.26 **
CV	−0.01	0.06	−0.03
MAGE	−0.03	0.03	0.08
MAG	0.21 **	0.29 **	0.16 **
LI	0.10 *	0.17 *	0.06

Spearman’s rank correlation coefficients (r) are presented. Significant coefficients are highlighted in bold: * *p* < 0.05; ** *p* < 0.01. Abbreviations: CGM, continuous glucose monitoring; CV, Coefficient of Variation; eGDR, estimated glucose disposal rate; LI, Lability Index; MAG, Mean Absolute Glucose rate of change; MAGE, Mean Amplitude of Glycemic Excursions; TAR L-1, Time Above Range, Level 1; TAR L-2, Time Above Range, Level 2; TBR L-1, Time Below Range, Level 1; TBR L-2, Time Below Range, Level 2; TIR, Time In the target Range; T1D, type 1 diabetes.

**Table 7:** Clinical and anthropometric predictors of CGM parameters in subjects with T1D in multivariate stepwise regression analysis (model set 1).

Parameter	Significant Predictors	R^2^	F	*p*
**Men**				
TIR	WC (β = 0.552), eGDR (β = 0.353)	0.15	7.73	0.0009
TAR L-1	WC (β = −0.542), eGDR (β = −0.334)	0.11	5.36	0.002
TAR L-2	WC (β = −0.37), eGDR (β = −0.325)	0.17	3.57	0.001
TBR L-2	eGDR (β = 0.321)	0.06	2.74	0.046
MAGE	WC (β = −0.539), eGDR (β = −0.275)	0.16	12.09	0.0002
MAG	Daily insulin dose (β = 0.244)	0.15	5.34	0.0005
LI	WC (β = −0.409)	0.16	8.28	0.0004
**Women**				
TIR	Age (β = −0.16), BMI (β = 0.285), eGDR (β = 0.25)	0.08	9.92	<0.0001
TAR L-1	eGDR (β = −0.264)	0.08	2.76	0.009
TAR L-2	Age (β = −0.45), BMI (β = 0.285), eGDR (β = 0.25)	0.15	5.85	0.0002
TBR L-1	eGDR (β = 0.273), eGFR (β = 0.174)	0.11	4.19	0.0002
CV	BMI (β = 0.188), eGFR (β = −0.233)	0.06	3.05	0.01
MAGE	BMI (β = −0.261), eGDR (β = −0.214), eGFR (β = 0.26)	0.21	15.02	0.0003
MAG	WC (β = −0.32)	0.19	6.36	0.003
LI	BMI (β = −0.299), eGDR (β = −0.178), eGFR (β = 0.327)	0.11	7.11	<0.0001

Parameters of the models: β = beta coefficient, R^2^ = R-Squared (or the coefficient of determination), F = F-statistic; *p* = *p*-value. Abbreviations: BMI, body mass index; CGM, continuous glucose monitoring; CV, Coefficient of Variation; eGDR, estimated glucose disposal rate; eGFR, estimated glomerular filtration rate; LI, Lability Index; MAG, Mean Absolute Glucose rate of change; MAGE, Mean Amplitude of Glycemic Excursions; TAR L-1, Time Above Range, Level 1; TAR L-2, Time Above Range, Level 2; TBR L-1, Time Below Range, Level 1; TBR L-2, Time Below Range, Level 2; TIR, Time In the target Range; T1D, type 1 diabetes; WC, waist circumference.

**Table 8:** Clinical and body composition parameters as predictors of CGM metrics in subjects with T1D in multivariate stepwise regression analysis (model set 2).

Parameter	Significant Predictors	R^2^	F	*p*
**Men**				
TIR	Daily insulin dose (β = −0.31), total fat mass (β = 0.263)	0.17	5.95	0.004
TAR L-1	Daily insulin dose (β = 0.265)	0.15	3.32	0.03
TAR L-2	Daily insulin dose (β = 0.301), total fat mass (β = −0.36)	0.37	8.00	<0.0001
CV	Android fat mass (β = −0.34)	0.11	7.66	0.007
MAGE	Trunk fat mass (β = −2.1), android fat mass (β = −1.44)	0.32	8.76	<0.0001
MAG	eGDR (β = 0.444)	0.13	2.8	0.048
LI	Total fat mass (β = −1.7)	0.27	5.55	0.0007
**Women**				
TIR	Gynoid fat mass (β = −0.46)	0.15	2.45	0.04
TAR L-1	Gynoid fat mass (β = 0.378)	0.15	2.50	0.04
TAR L-2	Gynoid fat mass (β = 0.573), eGDR (β = −0.36)	0.20	2.06	0.04
TBR L-1	Daily insulin dose (β = 0.310)	0.14	4.47	0.02
TBR L-2	eGDR (β = 0.413)	0.16	3.53	0.02
CV	Daily insulin dose (β = 0.346)	0.19	6.32	0.003
LI	eGFR (β = 0.652)	0.28	5.24	0.001

Parameters of the models: β = beta coefficient, R^2^ = R-Squared (or the coefficient of determination), F = F-statistic; *p* = *p*-value. Abbreviations: CGM, continuous glucose monitoring; CV, Coefficient of Variation; eGDR, estimated glucose disposal rate; eGFR, estimated glomerular filtration rate; LI, Lability Index; MAG, Mean Absolute Glucose rate of change; MAGE, Mean Amplitude of Glycemic Excursions; TAR L-1, Time Above Range, Level 1; TAR L-2, Time Above Range, Level 2; TBR L-1, Time Below Range, Level 1; TBR L-2, Time Below Range, Level 2; TIR, Time In the target Range; T1D, type 1 diabetes.

## Data Availability

The source data are available from the corresponding authors upon reasonable request.
